# Crystal structures of hydrogen-bonded co-crystals as liquid crystal precursors: 4-(*n*-pent­yloxy)benzoic acid–(*E*)-1,2-bis­(pyridin-4-yl)ethene (2/1) and 4-(*n*-hex­yloxy)benzoic acid–(*E*)-1,2-bis­(pyridin-4-yl)ethene (2/1)

**DOI:** 10.1107/S2056989016017655

**Published:** 2016-11-08

**Authors:** Yohei Tabuchi, Kazuma Gotoh, Hiroyuki Ishida

**Affiliations:** a Department of Chemistry, Faculty of Science, Okayama University, Okayama 700-8530, Japan

**Keywords:** crystal structure, (*E*)-1,2-bis­(pyridin-4-yl)ethene, 4-(*n*-pent­yloxy)benzoic acid, 4-(*n*-hex­yloxy)benzoic acid hydrogen-bonded liquid crystal

## Abstract

The crystal structures of title hydrogen-bonded co-crystals, 2C_12_H_16_O_3_·C_12_H_10_N_2_, (I), and 2C_13_H_18_O_3_·C_12_H_10_N_2_, (II), have been determined at 93 K. In (I), the asymmetric unit consists of one 4-(*n*-pent­yloxy)benzoic acid mol­ecule and one half-mol­ecule of (*E*)-1,2-bis­(pyridin-4-yl)ethene, which lies about an inversion centre. The asymmetric unit of (II) comprises two crystallographically independent 4-(*n*-hex­yloxy)benzoic acid mol­ecules and one 1,2-bis­(pyridin-4-yl)ethene mol­ecule. In each crystal, the acid and base components are linked by O—H⋯N hydrogen bonds, forming a linear hydrogen-bonded 2:1 unit of the acid and the base. The 2:1 units are linked *via* C—H⋯π and π–π inter­actions [centroid–centroid distances of 3.661 (2) and 3.909 (2) Å for (I), and 3.546 (2)–3.725 (4) Å for (II)], forming column structures. In (II), the base mol­ecule is orientationally disordered over two sets of sites approximately around the N⋯N mol­ecular axis, with an occupancy ratio of 0.647 (4):0.353 (4), and the average structure of the 2:1 unit adopts nearly pseudo-*C*_2_ symmetry. Both compounds show liquid-crystal behaviour.

## Abstract 2

The two title compounds comprise two acid mol­ecules and one base mol­ecule linked by O—H⋯N hydrogen bonds, forming a linear hydrogen-bonded 2:1 unit.

## Chemical context   

Co-crystals of 4-alk­oxy­benzoic acid [CH_3_(CH_2_)_*n*_OC_6_H_4_CO_2_H, *n* = 0–9]–4,4′-bipyridyl (2/1), 4-alk­oxy­benzoic acid [CH_3_(CH_2_)_*n*_OC_6_H_4_CO_2_H, *n* = 0–9]–(*E*)-1,2-bis­(pyridin-4-yl)ethene [common name: *trans*-1,2-bis­(4-pyrid­yl)ethyl­ene] (2/1) and 4-alkyl­benzoic acid [CH_3_(CH_2_)_*n*_C_6_H_4_CO_2_H, *n* = 3, 4, 7]–(*E*)-1,2-bis­(pyridin-4-yl)ethene (2/1), in which the two acid mol­ecules and the base mol­ecule are held together through inter­molecular hydrogen bonds, show thermotropic liquid crystallinity (Kato *et al.*, 1990 ▸, 1993 ▸). Of these co-crystals, crystal structures of 4,4′-bipyridyl with 4-meth­oxy­benzoic acid (Mukherjee & Desiraju, 2014 ▸; Ramon *et al.*, 2014 ▸), 4-eth­oxy-, 4-*n*-prop­oxy- and 4-*n*-but­oxy­benzoic acid (Tabuchi *et al.*, 2015*a* ▸) have been reported. Recently, the structures of (*E*)-1,2-bis­(pyridin-4-yl)ethene with 4-meth­oxy- 4-eth­oxy-, 4-*n*-prop­oxy- and 4-*n*-but­oxy­benzoic acid were also reported (Tabuchi *et al.*, 2016 ▸). As an expansion of our work on the structural characterization of the hydrogen-bonded co-crystals which exhibit liquid-crystal behaviour, we have prepared the title compounds and analysed their crystal structures.
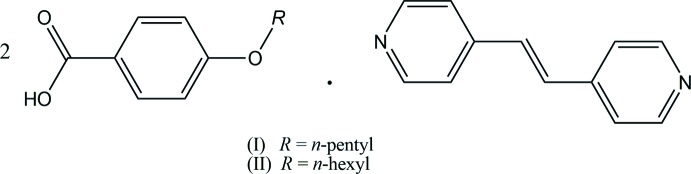


## Structural commentary   

**Figure 1:**
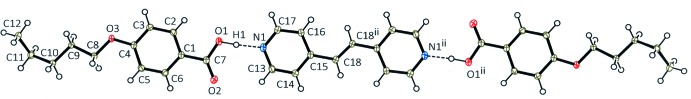
The mol­ecular structure of compound (I), showing the atom-numbering scheme. Displacement ellipsoids of non-H atoms are drawn at the 50% probability level and H atoms are shown as circles of arbitrary size. O—H⋯N hydrogen bonds are indicated by dashed lines. [Symmetry code: (ii) −*x* + 2, −*y* − 1, −*z*.]

The mol­ecular structures of compounds (I) and (II) are shown in Figs. 1 ▸ and 2 ▸, respectively. The asymmetric unit of (I) consists of one 4-pentyl­oxybenzoic acid mol­ecule and one half-mol­ecule of (*E*)-1,2-bis­(pyridin-4-yl)ethene, which lies about an inversion centre. The two acid mol­ecules and the base mol­ecule are linked *via* O—H⋯N hydrogen bonds (Table 1 ▸) to afford a centrosymmetric linear 2:1 unit. The hydrogen-bonded asymmetric unit is essentially planar with dihedral angles of 1.98 (10), 2.00 (10) and 3.69 (4)°, respectively, between the pyridine N1/C13–C17 and carboxyl O1/C7/O2 planes, the carboxyl and benzene C1–C6 planes, and the pyridine and benzene rings, respectively. On the other hand, the terminal alkyl C9–C12 chain deviates from the benzoic acid plane and adopts a *gauche* conformation with a C9—C10—C11—C12 torsion angle of −65.22 (10)°.

**Figure 3:**
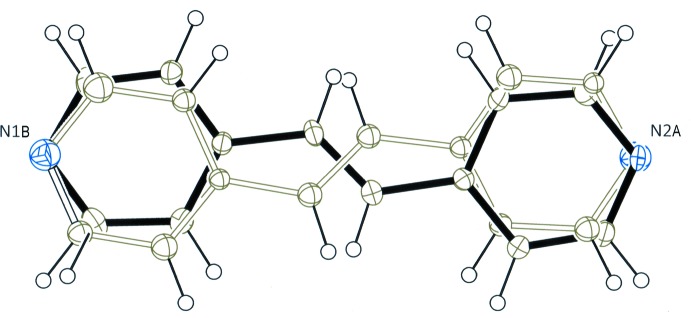
The disordered structure of the (*E*)-1,2-bis­(pyridin-4-yl)ethene mol­ecule in compound (I). The major and minor components are shown as solid and open bonds, respectively.

The asymmetric unit of (II) is composed of two crystallographically independent 4-hexyl­oxybenzoic acid mol­ecules and one (*E*)-1,2-bis­(pyridin-4-yl)ethene mol­ecule, and the two acids and the base are linked by O—H⋯N hydrogen bonds (Table 2 ▸), forming a linear hydrogen-bonded 2:1 aggregate with *trans*-zigzag alkyl chains. The base mol­ecule is orientationally disordered over two sets of sites approximately around the N⋯N long axis of the mol­ecule (Fig. 3 ▸), as also observed in the co-crystal of 4,4′-sulfonyl­diphenol–(*E*)-1,2-bis­(pyridin-4-yl)ethene (1/1) (Ferguson *et al.*, 1999 ▸). Similar orientational disorder has been observed in the crystals of stilbene and azo­benzene (Harada & Ogawa, 2004 ▸). The occupancy of the two components was refined to 0.647 (4) and 0.353 (4). Both the major and minor components of the base mol­ecule are approximately planar with dihedral angles of 8.0 (2) and 7.0 (5)°, respectively, between the two pyridine rings in each component. The two independent acid mol­ecules are also approximately planar. The maximum deviation from the mean plane of O1–O3/C1–C13 is 0.1530 (9) Å at atom O2, and that from the plane of O4–O6/C14–C26 is 0.1336 (9) Å at atom O4. The dihedral angles between the O1/C7/O2 and C1–C6 planes and between the O4/C20/O5 and C14–C19 planes are 8.57 (14) and 3.66 (14)°, respectively. The benzene C1–C6 ring is essentially coplanar with the adjacent hydrogen-bonded pyridine N1*A*/C27*A*–C31*A* (N1*B*/C27*B*–C31*B*) ring and makes dihedral angles of 0.14 (16) and 0.8 (3)° with the major and minor components, respectively. On the other hand, the benzene C14–C19 ring and the pyridine N2*A*/C32*A*–C36*A* (N2*B*/C32*B*–C36*B*) ring are inclined slightly to one another by 9.60 (17) and 10.1 (3)° for the major and minor components, respectively.

The 2:1 unit of the acid and the base of (I) adopts inversion symmetry, as observed for those in 4-meth­oxy­benzoic acid–(*E*)-1,2-bis­(pyridin-4-yl)ethene (2/1) and 4-*n*-but­oxy­benzoic acid–1,2-bis­(pyridin-4-yl)ethene (2/1) (Tabuchi *et al.*, 2016 ▸), while the average structure of the 2:1 unit of (II) shows nearly pseudo-*C*_2_ symmetry around an axis passing through the midpoint of the N⋯N mol­ecular axis of 1,2-bis­(pyridin-4-yl)ethene.

## Supra­molecular features   

**Figure 4:**
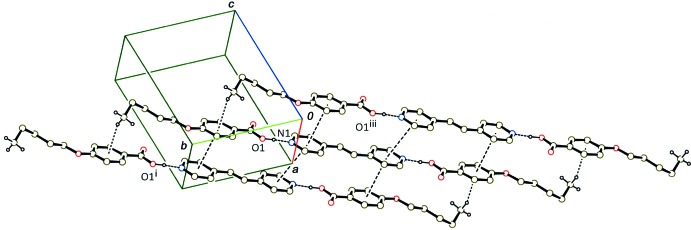
A partial packing diagram of compound (I), showing a column structure formed by C—H⋯π and π–π stacking inter­actions (dashed lines). H atoms except for the hy­droxy and methyl groups have been omitted. [Symmetry codes: (i) *x*, *y* + 1, *z*; (iii) *x*, *y* − 1, *z*.]

In the crystal of (I), the 2:1 units are stacked into a column along the *b* axis through a C—H⋯π inter­action between the methyl group and the benzene ring (Table 1 ▸) and π–π inter­actions between the benzene and pyridine rings and between the pyridine rings (Fig. 4 ▸) in a similar manner to the 2:1 units in 4-*n*-but­oxy­benzoic acid–1,2-bis­(pyridin-4-yl)ethene (2/1) (Tabuchi *et al.*, 2016 ▸). The centroid–centroid distances are 3.661 (2) and 3.909 (2) Å, respectively, between the benzene and pyridine rings and between the pyridine rings. Arrangements of the columns of the 2:1 units in (I) and 4-*n*-but­oxy­benzoic acid–1,2-bis­(pyridin-4-yl)ethene (2/1) are also quite similar to each other.

**Figure 5:**
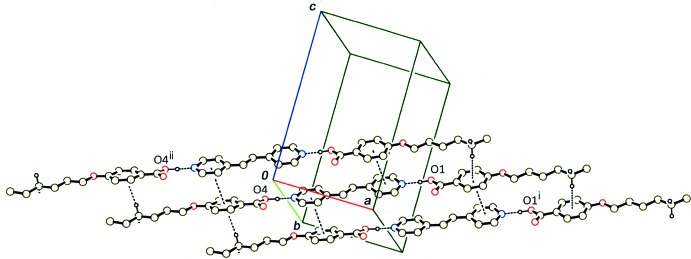
A partial packing diagram of compound (II), showing a column structure formed by C—H⋯π and π–π stacking inter­actions (dashed lines). H atoms except for the hy­droxy and methyl­ene groups involved in the inter­molecular inter­actions have been omitted. [Symmetry codes: (i) *x* + 1, *y*, *z*; (ii) *x* − 1, *y*, *z*.]

In the crystal of (II), the 2:1 units are stacked into a column along the *a* axis through C—H⋯π inter­actions between the methyl­ene groups and the benzene rings (Table 1 ▸) and π–π inter­actions between the benzene rings and the pyridine rings (Fig. 5 ▸). The centroid–centroid distances are 3.546 (2), 3.662 (4), 3.652 (2) and 3.725 (4) Å, respectively, between the benzene C1–C6 and pyridine N1*A*/C27*A*–C31*A* rings, the benzene C1–C6 and pyridine N1*B*/C27*B*–C31*B* rings, the benzene C14–C19 and pyridine N2*A*/C32*A*–C36*A* rings, and the benzene C14–C19 and pyridine N2*B*/C32*B*–C36*B* rings.

## Database survey   

A search of the Cambridge Structural Database (Version 5.37, last update May 2016; Groom *et al.*, 2016 ▸) for organic co-crystals of 1,2-bis­(pyridin-4-yl)ethene gave eight structures that exhibit orientational disorder of the 1,2-bis­(pyridin-4-yl)ethane mol­ecule around the long mol­ecular axis (Refcodes: APEDOP, AWEYAD, EWOGUM, IKUJED, LIPXAJ, MOBZIM, SEDYAC, OKIGOG). Crystal structures of similar co-crystals of 4-alk­oxy­benzoic acid–bipyridyl derivative (2/1), which show thermotropic liquid crystallinity, namely, (*E*)-1,2-bis­(pyridin-4-yl)ethane with 4-meth­oxy­benzoic acid (Mukherjee & Desiraju, 2014 ▸), (*E*)-1,2-bis­(pyridin-4-yl)ethane with 4-eth­oxy-, 4-*n*-prop­oxy- and 4-*n*-but­oxy­benzoic acid (Tabuchi *et al.*, 2015*b* ▸) have been reported.

## Synthesis and crystallization   

Single crystals of compounds (I) and (II) were obtained from ethanol solutions of (*E*)-1,2-bis­(pyridin-4-yl)ethene with 4-(*n*-pent­yloxy)benzoic acid and 4-(*n*-hex­yloxy)benzoic acid, respectively, at room temperature [ethanol solution (180 ml) of 1,2-bis­(pyridin-4-yl)ethene (57 mg) and 4-(*n*-pent­yloxy)benzoic acid (130 mg) for (I), and ethanol solution (180 ml) of 1,2-bis­(pyridin-4-yl)ethene (53 mg) and 4-(*n*-hex­yloxy)benzoic acid (130 mg) for (II)].

## Phase transitions   

Phase transitions for compounds (I) and (II) were observed by DSC and the liquid crystal phases were confirmed by polarizing microscope. DSC measurements were performed by using a Perkin Elmer Pyris 1 in the temperature range from 110 K to the melting temperature at a heating rate of 10 K min^−1^. In addition, for compound (I) DSC was carried out in the range of 420–450 K at a rate of 0.5 K min^−1^ to determine the transition temperatures and enthalpies of two successive phase transitions. Phase transition temperatures (K) and enthalpies (kJ mol^−1^) obtained by DSC are as follows:

(I) 384.8 (4) [21.7 (7)] K_1_ → K_2_, 401 (1) [31 (3)] K_2_→ S_A_, 445.3 (4) [3.7 (4)] S_A_ → N, 446.4 (4) [4.5 (3)] N → I.

(II) 412.5 (8) [46 (3)] K → S_A_, 449.2 (4) [16.3 (7)] S_A_ → I.

K, S_A_, N and I denote crystal, smectic A, nematic and isotropic phases, respectively. The observed transition temperatures and enthalpies are good agreement with the reported values (Kato *et al.*, 1993 ▸).

## Refinement   

Crystal data, data collection and structure refinement details are summarized in Table 3 ▸. For both compounds, C-bound H atoms were positioned geometrically with C—H = 0.95–0.99 Å and were refined as riding with *U*_iso_(H) = 1.2*U*_eq_(C) or 1.5*U*_eq_(methyl C). The O-bound H atoms were located in a difference Fourier map and refined freely [refined O—H = 1.02 (2) Å for (I), and 0.99 (2) and 1.09 (2) Å for (II)]. In (II), the 1,2-bis­(pyridin-4-yl)ethene mol­ecule was found to be disordered over two sets of sites in the difference Fourier map and the occupancy of the two components was refined to 0.647 (4) and 0.353 (4). For the minor component, C and N atoms were refined isotropically to avoid undesirable displace­ment ellipsoids. The geometry of the pyridine rings of the minor component was restrained to be similar to that of the major one using a *SAME* instruction.

## Supplementary Material

Crystal structure: contains datablock(s) General, I, II. DOI: 10.1107/S2056989016017655/hb7630sup1.cif

Structure factors: contains datablock(s) I. DOI: 10.1107/S2056989016017655/hb7630Isup2.hkl

Structure factors: contains datablock(s) II. DOI: 10.1107/S2056989016017655/hb7630IIsup3.hkl

Supporting information file. DOI: 10.1107/S2056989016017655/hb7630Isup4.cml

Supporting information file. DOI: 10.1107/S2056989016017655/hb7630IIsup5.cml

CCDC references: 1515164, 1515165

Additional supporting information:  crystallographic information; 3D view; checkCIF report

## Figures and Tables

**Figure 2:**
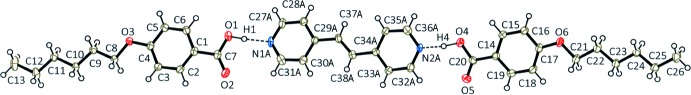
The mol­ecular structure of compound (II), showing the atom-numbering scheme. Displacement ellipsoids of non-H atoms are drawn at the 50% probability level and H atoms are shown as circles of arbitrary size. O—H⋯N hydrogen bonds are indicated by dashed lines. For the disordered base mol­ecule, only the major component is shown.

**Table 1:** Hydrogen-bond geometry (Å, °) for (I) *Cg*1 is the centroid of the benzene C1–C6 ring.

*D*—H⋯*A*	*D*—H	H⋯*A*	*D*⋯*A*	*D*—H⋯*A*
O1—H1⋯N1	1.016 (19)	1.580 (19)	2.5936 (17)	175 (2)
C12—H12*A*⋯*Cg*1^i^	0.98	2.64	3.592 (2)	151

Symmetry code: (i) 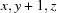.

**Table 2:** Hydrogen-bond geometry (Å, °) for (II) *Cg*1 and *Cg*2 are the centroids of the benzene C1–C6 and C14–C19 rings, respectively.

*D*—H⋯*A*	*D*—H	H⋯*A*	*D*⋯*A*	*D*—H⋯*A*
O1—H1⋯N1*A*	0.99 (2)	1.65 (2)	2.635 (5)	176.5 (15)
O1—H1⋯N1*B*	0.99 (2)	1.63 (2)	2.616 (14)	176.3 (19)
O4—H4⋯N2*A*	1.08 (3)	1.51 (3)	2.584 (6)	172.8 (18)
O4—H4⋯N2*B*	1.08 (3)	1.54 (3)	2.618 (15)	172.5 (18)
C12—H12*A*⋯*Cg*1^i^	0.99	2.99	3.720 (2)	132
C24—H24*A*⋯*Cg*2^ii^	0.99	2.93	3.838 (2)	154

Symmetry codes: (i) 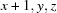; (ii) 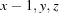.

**Table 3:** Experimental details

	(I)	(II)
Crystal data		
Chemical formula	2C_12_H_16_O_3_·C_12_H_10_N_2_	2C_13_H_18_O_3_·C_12_H_10_N_2_
*M*_r_	598.74	626.79
Crystal system, space group	Triclinic, *P* 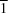	Triclinic, *P* 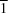
Temperature (K)	93	93
*a*, *b*, *c* (Å)	7.406 (4), 9.042 (4), 11.719 (5)	9.107 (3), 12.020 (5), 16.672 (6)
α, β, γ (°)	80.420 (17), 81.03 (2), 87.66 (3)	81.584 (16), 88.416 (15), 67.905 (15)
*V* (Å^3^)	764.2 (6)	1672.2 (11)
*Z*	1	2
Radiation type	Mo *K*α	Mo *K*α
μ (mm^−1^)	0.09	0.08
Crystal size (mm)	0.45 × 0.28 × 0.10	0.55 × 0.24 × 0.07

Data collection		
Diffractometer	Rigaku R-AXIS RAPIDII	Rigaku R-AXIS RAPIDII
Absorption correction	Multi-scan (*ABSCOR*; Higashi, 1995 ▸)	Multi-scan (*ABSCOR*; Higashi, 1995 ▸)
*T*_min_, *T*_max_	0.789, 0.991	0.819, 0.994
No. of measured, independent and observed [*I* > 2σ(*I*)] reflections	9720, 3500, 3146	16796, 7635, 5611
*R*_int_	0.022	0.031
(sin θ/λ)_max_ (Å^−1^)	0.649	0.649

Refinement		
*R*[*F* ^2^ > 2σ(*F* ^2^)], *wR*(*F* ^2^), *S*	0.042, 0.118, 1.08	0.043, 0.110, 1.01
No. of reflections	3500	7635
No. of parameters	204	482
No. of restraints	0	24
H-atom treatment	H atoms treated by a mixture of independent and constrained refinement	H atoms treated by a mixture of independent and constrained refinement
Δρ_max_, Δρ_min_ (e Å^−3^)	0.22, −0.49	0.25, −0.29

Computer programs: *RAPID-AUTO* (Rigaku, 2006 ▸), *Il Milione* (Burla *et al.*, 2007 ▸), *SHELXL2014* (Sheldrick, 2015 ▸), *ORTEP-3 for Windows* (Farrugia, 2012 ▸), *CrystalStructure* (Rigaku, 2010 ▸) and *PLATON* (Spek, 2015 ▸).

## supplementary crystallographic information

### (I) 4-(n-Pentyloxy)benzoic acid–(E)-1,2-bis(pyridin-4-yl)ethene (2/1) Crystal data

**Table:**

2C_12_H_16_O_3_·C_12_H_10_N_2_	*Z* = 1
*M**_r_* = 598.74	*F*(000) = 320.00
Triclinic, *P*1	*D*_x_ = 1.301 Mg m^−^^3^
*a* = 7.406 (4) Å	Mo *K*α radiation, λ = 0.71075 Å
*b* = 9.042 (4) Å	Cell parameters from 10871 reflections
*c* = 11.719 (5) Å	θ = 3.1–30.0°
α = 80.420 (17)°	µ = 0.09 mm^−^^1^
β = 81.03 (2)°	*T* = 93 K
γ = 87.66 (3)°	Block, colorless
*V* = 764.2 (6) Å^3^	0.45 × 0.28 × 0.10 mm

### (I) 4-(n-Pentyloxy)benzoic acid–(E)-1,2-bis(pyridin-4-yl)ethene (2/1) Data collection

**Table:**

Rigaku R-AXIS RAPIDII diffractometer	3146 reflections with *I* > 2σ(*I*)
Detector resolution: 10.000 pixels mm^-1^	*R*_int_ = 0.022
ω scans	θ_max_ = 27.5°, θ_min_ = 3.1°
Absorption correction: multi-scan (*ABSCOR*; Higashi, 1995)	*h* = −9→9
*T*_min_ = 0.789, *T*_max_ = 0.991	*k* = −11→11
9720 measured reflections	*l* = −15→15
3500 independent reflections	

### (I) 4-(n-Pentyloxy)benzoic acid–(E)-1,2-bis(pyridin-4-yl)ethene (2/1) Refinement

**Table:**

Refinement on *F*^2^	Primary atom site location: structure-invariant direct methods
Least-squares matrix: full	Secondary atom site location: difference Fourier map
*R*[*F*^2^ > 2σ(*F*^2^)] = 0.042	Hydrogen site location: mixed
*wR*(*F*^2^) = 0.118	H atoms treated by a mixture of independent and constrained refinement
*S* = 1.08	*w* = 1/[σ^2^(*F*_o_^2^) + (0.0823*P*)^2^ + 0.0761*P*] where *P* = (*F*_o_^2^ + 2*F*_c_^2^)/3
3500 reflections	(Δ/σ)_max_ = 0.001
204 parameters	Δρ_max_ = 0.22 e Å^−^^3^
0 restraints	Δρ_min_ = −0.49 e Å^−^^3^

### (I) 4-(n-Pentyloxy)benzoic acid–(E)-1,2-bis(pyridin-4-yl)ethene (2/1) Special details

**Table:**

Geometry. All esds (except the esd in the dihedral angle between two l.s. planes) are estimated using the full covariance matrix. The cell esds are taken into account individually in the estimation of esds in distances, angles and torsion angles; correlations between esds in cell parameters are only used when they are defined by crystal symmetry. An approximate (isotropic) treatment of cell esds is used for estimating esds involving l.s. planes.
Refinement. Reflections were merged by SHELXL according to the crystal class for the calculation of statistics and refinement. _reflns_Friedel_fraction is defined as the number of unique Friedel pairs measured divided by the number that would be possible theoretically, ignoring centric projections and systematic absences.

### (I) 4-(n-Pentyloxy)benzoic acid–(E)-1,2-bis(pyridin-4-yl)ethene (2/1) Fractional atomic coordinates and isotropic or equivalent isotropic displacement parameters (Å^2^)

**Table:**

	*x*	*y*	*z*	*U*_iso_*/*U*_eq_	
O1	0.63171 (10)	0.22291 (7)	0.13060 (6)	0.02355 (18)	
H1	0.689 (3)	0.121 (2)	0.1206 (19)	0.092 (7)*	
O2	0.74301 (10)	0.19377 (7)	0.30027 (6)	0.02484 (18)	
O3	0.35890 (9)	0.84595 (7)	0.25941 (5)	0.01847 (16)	
N1	0.77776 (10)	−0.03371 (8)	0.09355 (7)	0.01817 (18)	
C1	0.58287 (11)	0.42040 (9)	0.23882 (7)	0.01524 (19)	
C2	0.48572 (12)	0.49788 (9)	0.15403 (7)	0.01714 (19)	
H2	0.4677	0.4519	0.0895	0.021*	
C3	0.41522 (12)	0.64028 (9)	0.16208 (7)	0.01730 (19)	
H3	0.3515	0.6923	0.1028	0.021*	
C4	0.43815 (11)	0.70746 (9)	0.25794 (7)	0.01525 (19)	
C5	0.53474 (11)	0.63145 (9)	0.34369 (7)	0.01655 (19)	
H5	0.5515	0.6769	0.4087	0.020*	
C6	0.60648 (11)	0.48861 (9)	0.33337 (7)	0.01627 (19)	
H6	0.6724	0.4371	0.3917	0.020*	
C7	0.66150 (11)	0.26796 (9)	0.22765 (7)	0.01667 (19)	
C8	0.37285 (12)	0.91903 (9)	0.35775 (7)	0.01651 (19)	
H8A	0.4999	0.9515	0.3542	0.020*	
H8B	0.3389	0.8490	0.4319	0.020*	
C9	0.24406 (12)	1.05385 (9)	0.35257 (7)	0.01660 (19)	
H9A	0.1189	1.0215	0.3500	0.020*	
H9B	0.2833	1.1261	0.2805	0.020*	
C10	0.24376 (12)	1.12976 (9)	0.45997 (7)	0.01841 (19)	
H10A	0.2339	1.0516	0.5306	0.022*	
H10B	0.3621	1.1803	0.4527	0.022*	
C11	0.08889 (12)	1.24497 (10)	0.47705 (7)	0.0202 (2)	
H11A	−0.0292	1.1953	0.4812	0.024*	
H11B	0.0897	1.2793	0.5529	0.024*	
C12	0.10103 (12)	1.38069 (10)	0.38101 (8)	0.0223 (2)	
H12A	0.2191	1.4291	0.3745	0.033*	
H12B	0.0019	1.4519	0.4000	0.033*	
H12C	0.0899	1.3489	0.3065	0.033*	
C13	0.86824 (12)	−0.11635 (10)	0.17238 (8)	0.01939 (19)	
H13	0.8816	−0.0775	0.2411	0.023*	
C14	0.94346 (12)	−0.25609 (10)	0.15845 (7)	0.01845 (19)	
H14	1.0067	−0.3113	0.2168	0.022*	
C15	0.92594 (11)	−0.31554 (9)	0.05815 (7)	0.01598 (19)	
C16	0.83293 (13)	−0.22774 (10)	−0.02441 (8)	0.0201 (2)	
H16	0.8189	−0.2629	−0.0945	0.024*	
C17	0.76135 (13)	−0.08941 (10)	−0.00375 (8)	0.0206 (2)	
H17	0.6979	−0.0313	−0.0607	0.025*	
C18	1.00375 (12)	−0.46469 (9)	0.04498 (7)	0.0182 (2)	
H18	1.0649	−0.5152	0.1061	0.022*	

### (I) 4-(n-Pentyloxy)benzoic acid–(E)-1,2-bis(pyridin-4-yl)ethene (2/1) Atomic displacement parameters (Å^2^)

**Table:**

	*U*^11^	*U*^22^	*U*^33^	*U*^12^	*U*^13^	*U*^23^
O1	0.0347 (4)	0.0172 (3)	0.0210 (3)	0.0088 (3)	−0.0082 (3)	−0.0081 (3)
O2	0.0320 (4)	0.0177 (3)	0.0273 (4)	0.0074 (3)	−0.0124 (3)	−0.0053 (3)
O3	0.0253 (3)	0.0128 (3)	0.0193 (3)	0.0061 (2)	−0.0075 (2)	−0.0059 (2)
N1	0.0201 (4)	0.0134 (3)	0.0208 (4)	0.0013 (3)	−0.0010 (3)	−0.0044 (3)
C1	0.0156 (4)	0.0131 (4)	0.0162 (4)	−0.0001 (3)	0.0001 (3)	−0.0025 (3)
C2	0.0220 (4)	0.0160 (4)	0.0133 (4)	0.0007 (3)	−0.0012 (3)	−0.0040 (3)
C3	0.0213 (4)	0.0156 (4)	0.0148 (4)	0.0026 (3)	−0.0039 (3)	−0.0017 (3)
C4	0.0158 (4)	0.0121 (4)	0.0173 (4)	0.0005 (3)	−0.0007 (3)	−0.0026 (3)
C5	0.0175 (4)	0.0162 (4)	0.0175 (4)	0.0003 (3)	−0.0041 (3)	−0.0057 (3)
C6	0.0157 (4)	0.0156 (4)	0.0177 (4)	0.0008 (3)	−0.0039 (3)	−0.0021 (3)
C7	0.0173 (4)	0.0146 (4)	0.0175 (4)	0.0000 (3)	−0.0006 (3)	−0.0031 (3)
C8	0.0193 (4)	0.0139 (4)	0.0177 (4)	0.0024 (3)	−0.0051 (3)	−0.0050 (3)
C9	0.0196 (4)	0.0128 (4)	0.0185 (4)	0.0037 (3)	−0.0057 (3)	−0.0043 (3)
C10	0.0238 (4)	0.0150 (4)	0.0174 (4)	0.0047 (3)	−0.0056 (3)	−0.0043 (3)
C11	0.0230 (4)	0.0175 (4)	0.0191 (4)	0.0039 (3)	0.0001 (3)	−0.0043 (3)
C12	0.0210 (4)	0.0166 (4)	0.0274 (5)	0.0045 (3)	−0.0010 (3)	−0.0023 (3)
C13	0.0225 (4)	0.0178 (4)	0.0190 (4)	0.0012 (3)	−0.0018 (3)	−0.0077 (3)
C14	0.0209 (4)	0.0173 (4)	0.0178 (4)	0.0032 (3)	−0.0047 (3)	−0.0037 (3)
C15	0.0168 (4)	0.0133 (4)	0.0176 (4)	0.0007 (3)	−0.0008 (3)	−0.0036 (3)
C16	0.0274 (5)	0.0153 (4)	0.0192 (4)	0.0038 (3)	−0.0065 (3)	−0.0058 (3)
C17	0.0260 (5)	0.0148 (4)	0.0215 (4)	0.0042 (3)	−0.0057 (3)	−0.0035 (3)
C18	0.0224 (4)	0.0135 (4)	0.0193 (4)	0.0044 (3)	−0.0052 (3)	−0.0031 (3)

### (I) 4-(n-Pentyloxy)benzoic acid–(E)-1,2-bis(pyridin-4-yl)ethene (2/1) Geometric parameters (Å, º)

**Table:**

O1—C7	1.3220 (11)	C9—H9A	0.9900
O1—H1	1.02 (2)	C9—H9B	0.9900
O2—C7	1.2147 (12)	C10—C11	1.5300 (13)
O3—C4	1.3626 (11)	C10—H10A	0.9900
O3—C8	1.4393 (10)	C10—H10B	0.9900
N1—C13	1.3353 (12)	C11—C12	1.5160 (13)
N1—C17	1.3446 (12)	C11—H11A	0.9900
C1—C6	1.3912 (12)	C11—H11B	0.9900
C1—C2	1.3948 (13)	C12—H12A	0.9800
C1—C7	1.4922 (12)	C12—H12B	0.9800
C2—C3	1.3819 (12)	C12—H12C	0.9800
C2—H2	0.9500	C13—C14	1.3843 (13)
C3—C4	1.3981 (12)	C13—H13	0.9500
C3—H3	0.9500	C14—C15	1.3967 (12)
C4—C5	1.3956 (13)	C14—H14	0.9500
C5—C6	1.3932 (12)	C15—C16	1.3957 (13)
C5—H5	0.9500	C15—C18	1.4672 (13)
C6—H6	0.9500	C16—C17	1.3822 (13)
C8—C9	1.5158 (12)	C16—H16	0.9500
C8—H8A	0.9900	C17—H17	0.9500
C8—H8B	0.9900	C18—C18^i^	1.3294 (17)
C9—C10	1.5302 (12)	C18—H18	0.9500
			
C7—O1—H1	113.0 (12)	C11—C10—C9	114.03 (7)
C4—O3—C8	117.75 (7)	C11—C10—H10A	108.7
C13—N1—C17	117.92 (8)	C9—C10—H10A	108.7
C6—C1—C2	118.70 (8)	C11—C10—H10B	108.7
C6—C1—C7	120.67 (8)	C9—C10—H10B	108.7
C2—C1—C7	120.62 (8)	H10A—C10—H10B	107.6
C3—C2—C1	121.24 (8)	C12—C11—C10	114.07 (8)
C3—C2—H2	119.4	C12—C11—H11A	108.7
C1—C2—H2	119.4	C10—C11—H11A	108.7
C2—C3—C4	119.68 (8)	C12—C11—H11B	108.7
C2—C3—H3	120.2	C10—C11—H11B	108.7
C4—C3—H3	120.2	H11A—C11—H11B	107.6
O3—C4—C5	125.07 (8)	C11—C12—H12A	109.5
O3—C4—C3	115.09 (7)	C11—C12—H12B	109.5
C5—C4—C3	119.84 (8)	H12A—C12—H12B	109.5
C6—C5—C4	119.63 (8)	C11—C12—H12C	109.5
C6—C5—H5	120.2	H12A—C12—H12C	109.5
C4—C5—H5	120.2	H12B—C12—H12C	109.5
C1—C6—C5	120.90 (8)	N1—C13—C14	122.91 (8)
C1—C6—H6	119.5	N1—C13—H13	118.5
C5—C6—H6	119.5	C14—C13—H13	118.5
O2—C7—O1	124.03 (8)	C13—C14—C15	119.64 (8)
O2—C7—C1	123.45 (8)	C13—C14—H14	120.2
O1—C7—C1	112.52 (8)	C15—C14—H14	120.2
O3—C8—C9	107.92 (7)	C16—C15—C14	117.09 (8)
O3—C8—H8A	110.1	C16—C15—C18	123.67 (8)
C9—C8—H8A	110.1	C14—C15—C18	119.25 (8)
O3—C8—H8B	110.1	C17—C16—C15	119.71 (8)
C9—C8—H8B	110.1	C17—C16—H16	120.1
H8A—C8—H8B	108.4	C15—C16—H16	120.1
C8—C9—C10	110.10 (7)	N1—C17—C16	122.73 (8)
C8—C9—H9A	109.6	N1—C17—H17	118.6
C10—C9—H9A	109.6	C16—C17—H17	118.6
C8—C9—H9B	109.6	C18^i^—C18—C15	125.75 (10)
C10—C9—H9B	109.6	C18^i^—C18—H18	117.1
H9A—C9—H9B	108.2	C15—C18—H18	117.1
			
C6—C1—C2—C3	0.67 (13)	C2—C1—C7—O1	1.72 (12)
C7—C1—C2—C3	−178.73 (7)	C4—O3—C8—C9	−168.61 (7)
C1—C2—C3—C4	−1.26 (13)	O3—C8—C9—C10	176.17 (6)
C8—O3—C4—C5	−1.08 (12)	C8—C9—C10—C11	−167.30 (7)
C8—O3—C4—C3	178.12 (7)	C9—C10—C11—C12	−65.22 (10)
C2—C3—C4—O3	−178.11 (7)	C17—N1—C13—C14	−0.60 (14)
C2—C3—C4—C5	1.13 (13)	N1—C13—C14—C15	0.09 (14)
O3—C4—C5—C6	178.72 (7)	C13—C14—C15—C16	0.60 (13)
C3—C4—C5—C6	−0.45 (13)	C13—C14—C15—C18	−179.09 (8)
C2—C1—C6—C5	0.03 (13)	C14—C15—C16—C17	−0.78 (14)
C7—C1—C6—C5	179.43 (7)	C18—C15—C16—C17	178.90 (8)
C4—C5—C6—C1	−0.14 (13)	C13—N1—C17—C16	0.40 (14)
C6—C1—C7—O2	2.64 (13)	C15—C16—C17—N1	0.30 (15)
C2—C1—C7—O2	−177.97 (8)	C16—C15—C18—C18^i^	−0.19 (18)
C6—C1—C7—O1	−177.67 (7)	C14—C15—C18—C18^i^	179.49 (11)

Symmetry code: (i) −*x*+2, −*y*−1, −*z*.

### (I) 4-(n-Pentyloxy)benzoic acid–(E)-1,2-bis(pyridin-4-yl)ethene (2/1) Hydrogen-bond geometry (Å, º)

*Cg*1 is the centroid of the benzene C1–C6 ring.

**Table:**

*D*—H···*A*	*D*—H	H···*A*	*D*···*A*	*D*—H···*A*
O1—H1···N1	1.016 (19)	1.580 (19)	2.5936 (17)	175 (2)
C12—H12*A*···*Cg*1^ii^	0.98	2.64	3.592 (2)	151

Symmetry code: (ii) *x*, *y*+1, *z*.

### (II) 4-(n-Hexyloxy)benzoic acid–(E)-1,2-bis(pyridin-4-yl)ethene Crystal data

**Table:**

2C_13_H_18_O_3_·C_12_H_10_N_2_	*Z* = 2
*M**_r_* = 626.79	*F*(000) = 672.00
Triclinic, *P*1	*D*_x_ = 1.245 Mg m^−^^3^
*a* = 9.107 (3) Å	Mo *K*α radiation, λ = 0.71075 Å
*b* = 12.020 (5) Å	Cell parameters from 15048 reflections
*c* = 16.672 (6) Å	θ = 3.3–30.0°
α = 81.584 (16)°	µ = 0.08 mm^−^^1^
β = 88.416 (15)°	*T* = 93 K
γ = 67.905 (15)°	Platelet, colorless
*V* = 1672.2 (11) Å^3^	0.55 × 0.24 × 0.07 mm

### (II) 4-(n-Hexyloxy)benzoic acid–(E)-1,2-bis(pyridin-4-yl)ethene Data collection

**Table:**

Rigaku R-AXIS RAPIDII diffractometer	5611 reflections with *I* > 2σ(*I*)
Detector resolution: 10.000 pixels mm^-1^	*R*_int_ = 0.031
ω scans	θ_max_ = 27.5°, θ_min_ = 3.3°
Absorption correction: multi-scan (*ABSCOR*; Higashi, 1995)	*h* = −11→11
*T*_min_ = 0.819, *T*_max_ = 0.994	*k* = −15→15
16796 measured reflections	*l* = −21→21
7635 independent reflections	

### (II) 4-(n-Hexyloxy)benzoic acid–(E)-1,2-bis(pyridin-4-yl)ethene Refinement

**Table:**

Refinement on *F*^2^	Primary atom site location: structure-invariant direct methods
Least-squares matrix: full	Secondary atom site location: difference Fourier map
*R*[*F*^2^ > 2σ(*F*^2^)] = 0.043	Hydrogen site location: mixed
*wR*(*F*^2^) = 0.110	H atoms treated by a mixture of independent and constrained refinement
*S* = 1.01	*w* = 1/[σ^2^(*F*_o_^2^) + (0.0639*P*)^2^] where *P* = (*F*_o_^2^ + 2*F*_c_^2^)/3
7635 reflections	(Δ/σ)_max_ = 0.001
482 parameters	Δρ_max_ = 0.25 e Å^−^^3^
24 restraints	Δρ_min_ = −0.29 e Å^−^^3^

### (II) 4-(n-Hexyloxy)benzoic acid–(E)-1,2-bis(pyridin-4-yl)ethene Special details

**Table:**

Geometry. All esds (except the esd in the dihedral angle between two l.s. planes) are estimated using the full covariance matrix. The cell esds are taken into account individually in the estimation of esds in distances, angles and torsion angles; correlations between esds in cell parameters are only used when they are defined by crystal symmetry. An approximate (isotropic) treatment of cell esds is used for estimating esds involving l.s. planes.
Refinement. Reflections were merged by SHELXL according to the crystal class for the calculation of statistics and refinement. _reflns_Friedel_fraction is defined as the number of unique Friedel pairs measured divided by the number that would be possible theoretically, ignoring centric projections and systematic absences.

### (II) 4-(n-Hexyloxy)benzoic acid–(E)-1,2-bis(pyridin-4-yl)ethene Fractional atomic coordinates and isotropic or equivalent isotropic displacement parameters (Å^2^)

**Table:**

	*x*	*y*	*z*	*U*_iso_*/*U*_eq_	Occ. (<1)
O1	1.42505 (10)	0.09185 (8)	0.27424 (5)	0.0333 (2)	
O2	1.54109 (10)	−0.04491 (8)	0.19145 (5)	0.0330 (2)	
O3	2.12484 (9)	−0.06159 (8)	0.40308 (5)	0.02806 (19)	
O4	−0.15865 (10)	0.35829 (8)	−0.05256 (5)	0.0309 (2)	
O5	−0.09887 (11)	0.20320 (9)	−0.12240 (6)	0.0434 (2)	
O6	−0.84790 (9)	0.48165 (8)	−0.18372 (5)	0.0302 (2)	
C1	1.70210 (13)	−0.00882 (10)	0.28693 (7)	0.0238 (2)	
C2	1.84421 (13)	−0.08040 (10)	0.25625 (7)	0.0241 (2)	
H2	1.8423	−0.1160	0.2092	0.029*	
C3	1.98902 (13)	−0.10090 (10)	0.29294 (7)	0.0239 (2)	
H3	2.0854	−0.1497	0.2713	0.029*	
C4	1.99056 (13)	−0.04879 (10)	0.36184 (7)	0.0237 (2)	
C5	1.84909 (13)	0.02307 (11)	0.39347 (7)	0.0279 (3)	
H5	1.8508	0.0581	0.4408	0.033*	
C6	1.70629 (13)	0.04319 (11)	0.35578 (7)	0.0267 (2)	
H6	1.6100	0.0930	0.3770	0.032*	
C7	1.54990 (14)	0.01020 (11)	0.24567 (7)	0.0257 (2)	
C8	2.27479 (13)	−0.13955 (11)	0.37704 (7)	0.0251 (2)	
H8A	2.2963	−0.1067	0.3219	0.030*	
H8B	2.2751	−0.2217	0.3759	0.030*	
C9	2.39862 (13)	−0.14475 (11)	0.43723 (7)	0.0278 (3)	
H9A	2.3721	−0.1744	0.4921	0.033*	
H9B	2.3957	−0.0616	0.4376	0.033*	
C10	2.56564 (12)	−0.22644 (11)	0.41889 (7)	0.0246 (2)	
H10A	2.5926	−0.1971	0.3640	0.029*	
H10B	2.5693	−0.3098	0.4189	0.029*	
C11	2.68871 (13)	−0.22971 (11)	0.48033 (7)	0.0275 (3)	
H11A	2.6810	−0.1458	0.4822	0.033*	
H11B	2.6643	−0.2626	0.5348	0.033*	
C12	2.85704 (14)	−0.30597 (13)	0.46084 (8)	0.0363 (3)	
H12A	2.8792	−0.2761	0.4051	0.044*	
H12B	2.8663	−0.3910	0.4622	0.044*	
C13	2.98061 (17)	−0.30353 (16)	0.51899 (10)	0.0512 (4)	
H13A	2.9707	−0.2195	0.5190	0.077*	
H13B	3.0867	−0.3511	0.5018	0.077*	
H13C	2.9643	−0.3385	0.5738	0.077*	
C14	−0.36643 (13)	0.34302 (10)	−0.12446 (6)	0.0224 (2)	
C15	−0.47394 (13)	0.44362 (10)	−0.09367 (6)	0.0229 (2)	
H15	−0.4367	0.4830	−0.0582	0.027*	
C16	−0.63337 (13)	0.48683 (10)	−0.11394 (7)	0.0242 (2)	
H16	−0.7056	0.5541	−0.0914	0.029*	
C17	−0.68806 (13)	0.43144 (10)	−0.16751 (7)	0.0247 (2)	
C18	−0.58315 (14)	0.33190 (11)	−0.19980 (7)	0.0274 (3)	
H18	−0.6201	0.2945	−0.2368	0.033*	
C19	−0.42317 (14)	0.28783 (11)	−0.17712 (7)	0.0265 (2)	
H19	−0.3514	0.2187	−0.1980	0.032*	
C20	−0.19545 (13)	0.29410 (11)	−0.10047 (7)	0.0253 (2)	
C21	−0.91312 (14)	0.43395 (12)	−0.24144 (7)	0.0313 (3)	
H21A	−0.8930	0.3473	−0.2229	0.038*	
H21B	−0.8647	0.4412	−0.2949	0.038*	
C22	−1.08930 (14)	0.50807 (11)	−0.24760 (7)	0.0295 (3)	
H22A	−1.1062	0.5951	−0.2607	0.035*	
H22B	−1.1358	0.4961	−0.1942	0.035*	
C23	−1.17552 (14)	0.47520 (11)	−0.31134 (7)	0.0292 (3)	
H23A	−1.1569	0.3878	−0.2990	0.035*	
H23B	−1.1310	0.4891	−0.3650	0.035*	
C24	−1.35357 (13)	0.54865 (11)	−0.31559 (7)	0.0264 (2)	
H24A	−1.3991	0.5308	−0.2630	0.032*	
H24B	−1.3720	0.6362	−0.3244	0.032*	
C25	−1.43892 (15)	0.52189 (12)	−0.38257 (8)	0.0337 (3)	
H25A	−1.4140	0.4334	−0.3760	0.040*	
H25B	−1.3987	0.5454	−0.4355	0.040*	
C26	−1.61824 (15)	0.58816 (13)	−0.38342 (9)	0.0421 (3)	
H26A	−1.6441	0.6759	−0.3899	0.063*	
H26B	−1.6598	0.5624	−0.3322	0.063*	
H26C	−1.6662	0.5688	−0.4287	0.063*	
N1A	1.1522 (5)	0.1162 (6)	0.2093 (3)	0.0228 (18)	0.647 (4)
C27A	1.0268 (6)	0.1865 (5)	0.2451 (4)	0.0282 (11)	0.647 (4)
H27A	1.0460	0.2167	0.2917	0.034*	0.647 (4)
C28A	0.8723 (3)	0.2193 (2)	0.22069 (19)	0.0263 (6)	0.647 (4)
H28A	0.7885	0.2697	0.2498	0.032*	0.647 (4)
C29A	0.8404 (2)	0.1776 (2)	0.15261 (17)	0.0218 (5)	0.647 (4)
C30A	0.9699 (4)	0.1041 (3)	0.11322 (15)	0.0288 (5)	0.647 (4)
H30A	0.9548	0.0733	0.0661	0.035*	0.647 (4)
C31A	1.1217 (5)	0.0767 (5)	0.1444 (3)	0.0334 (10)	0.647 (4)
H31A	1.2089	0.0263	0.1172	0.040*	0.647 (4)
N2A	0.1367 (6)	0.2855 (6)	−0.0092 (4)	0.0205 (17)	0.647 (4)
C32A	0.2515 (5)	0.2015 (4)	−0.0416 (2)	0.0273 (9)	0.647 (4)
H32A	0.2240	0.1651	−0.0826	0.033*	0.647 (4)
C33A	0.4095 (3)	0.1635 (2)	−0.01891 (17)	0.0253 (6)	0.647 (4)
H33A	0.4882	0.1045	−0.0453	0.030*	0.647 (4)
C34A	0.4525 (3)	0.2125 (2)	0.04299 (18)	0.0215 (5)	0.647 (4)
C35A	0.3292 (4)	0.2986 (3)	0.07937 (19)	0.0267 (7)	0.647 (4)
H35A	0.3510	0.3338	0.1228	0.032*	0.647 (4)
C36A	0.1757 (6)	0.3311 (7)	0.0507 (4)	0.0331 (14)	0.647 (4)
H36A	0.0931	0.3897	0.0755	0.040*	0.647 (4)
C37A	0.6747 (2)	0.21132 (16)	0.12689 (11)	0.0247 (5)	0.647 (4)
H37A	0.5972	0.2656	0.1569	0.030*	0.647 (4)
C38A	0.6201 (2)	0.17517 (17)	0.06689 (11)	0.0248 (5)	0.647 (4)
H38A	0.6960	0.1203	0.0367	0.030*	0.647 (4)
N1B	1.1539 (14)	0.1182 (14)	0.2089 (8)	0.042 (5)*	0.353 (4)
C27B	1.0102 (12)	0.1866 (12)	0.2366 (7)	0.039 (3)*	0.353 (4)
H27B	1.0050	0.2218	0.2845	0.046*	0.353 (4)
C28B	0.8728 (6)	0.2044 (5)	0.1947 (3)	0.0230 (12)*	0.353 (4)
H28B	0.7744	0.2562	0.2128	0.028*	0.353 (4)
C29B	0.8731 (6)	0.1498 (4)	0.1278 (3)	0.0188 (9)*	0.353 (4)
C30B	1.0202 (7)	0.0778 (5)	0.1017 (3)	0.0300 (14)*	0.353 (4)
H30B	1.0264	0.0383	0.0557	0.036*	0.353 (4)
C31B	1.1545 (9)	0.0643 (9)	0.1422 (5)	0.0271 (19)*	0.353 (4)
H31B	1.2536	0.0150	0.1232	0.033*	0.353 (4)
N2B	0.1398 (14)	0.2833 (15)	−0.0068 (9)	0.041 (5)*	0.353 (4)
C32B	0.2783 (10)	0.2036 (8)	−0.0344 (5)	0.027 (2)*	0.353 (4)
H32B	0.2733	0.1649	−0.0795	0.033*	0.353 (4)
C33B	0.4231 (6)	0.1788 (5)	0.0018 (3)	0.0224 (13)*	0.353 (4)
H33B	0.5160	0.1203	−0.0163	0.027*	0.353 (4)
C34B	0.4330 (7)	0.2395 (5)	0.0649 (3)	0.0199 (11)*	0.353 (4)
C35B	0.2947 (8)	0.3223 (6)	0.0889 (4)	0.0282 (17)*	0.353 (4)
H35B	0.2982	0.3655	0.1317	0.034*	0.353 (4)
C36B	0.1546 (12)	0.3436 (11)	0.0531 (6)	0.0187 (18)*	0.353 (4)
H36B	0.0621	0.4033	0.0706	0.022*	0.353 (4)
C37B	0.7297 (4)	0.1622 (3)	0.0844 (2)	0.0252 (10)*	0.353 (4)
H37B	0.7425	0.1243	0.0371	0.030*	0.353 (4)
C38B	0.5823 (5)	0.2222 (3)	0.1058 (2)	0.0248 (9)*	0.353 (4)
H38B	0.5724	0.2585	0.1536	0.030*	0.353 (4)
H1	1.325 (2)	0.0996 (18)	0.2481 (12)	0.079 (6)*	
H4	−0.033 (3)	0.3226 (19)	−0.0371 (12)	0.083 (6)*	

### (II) 4-(n-Hexyloxy)benzoic acid–(E)-1,2-bis(pyridin-4-yl)ethene Atomic displacement parameters (Å^2^)

**Table:**

	*U*^11^	*U*^22^	*U*^33^	*U*^12^	*U*^13^	*U*^23^
O1	0.0193 (4)	0.0407 (5)	0.0392 (5)	−0.0085 (4)	−0.0051 (4)	−0.0101 (4)
O2	0.0296 (5)	0.0326 (5)	0.0399 (5)	−0.0132 (4)	−0.0085 (4)	−0.0090 (4)
O3	0.0173 (4)	0.0357 (5)	0.0294 (4)	−0.0047 (4)	−0.0035 (3)	−0.0128 (3)
O4	0.0218 (4)	0.0338 (5)	0.0378 (5)	−0.0084 (4)	−0.0056 (3)	−0.0121 (4)
O5	0.0252 (5)	0.0484 (6)	0.0535 (6)	−0.0024 (4)	−0.0019 (4)	−0.0290 (5)
O6	0.0231 (4)	0.0317 (5)	0.0363 (4)	−0.0090 (4)	−0.0098 (3)	−0.0081 (4)
C1	0.0223 (6)	0.0238 (6)	0.0259 (5)	−0.0102 (5)	−0.0035 (4)	−0.0008 (4)
C2	0.0260 (6)	0.0221 (6)	0.0258 (5)	−0.0101 (5)	−0.0035 (4)	−0.0048 (4)
C3	0.0220 (6)	0.0220 (6)	0.0275 (5)	−0.0071 (5)	−0.0004 (4)	−0.0054 (4)
C4	0.0210 (5)	0.0259 (6)	0.0246 (5)	−0.0093 (5)	−0.0034 (4)	−0.0027 (4)
C5	0.0227 (6)	0.0343 (6)	0.0265 (5)	−0.0085 (5)	−0.0014 (5)	−0.0099 (5)
C6	0.0199 (5)	0.0311 (6)	0.0276 (6)	−0.0072 (5)	0.0001 (4)	−0.0060 (5)
C7	0.0240 (6)	0.0256 (6)	0.0292 (6)	−0.0122 (5)	−0.0034 (5)	−0.0003 (5)
C8	0.0181 (5)	0.0258 (6)	0.0293 (6)	−0.0044 (5)	−0.0017 (4)	−0.0074 (5)
C9	0.0211 (6)	0.0319 (6)	0.0283 (6)	−0.0060 (5)	−0.0024 (5)	−0.0077 (5)
C10	0.0195 (5)	0.0266 (6)	0.0266 (5)	−0.0070 (5)	−0.0028 (4)	−0.0046 (4)
C11	0.0229 (6)	0.0305 (6)	0.0284 (6)	−0.0094 (5)	−0.0059 (5)	−0.0022 (5)
C12	0.0217 (6)	0.0433 (8)	0.0390 (7)	−0.0087 (6)	−0.0051 (5)	0.0005 (6)
C13	0.0281 (7)	0.0636 (10)	0.0574 (9)	−0.0169 (7)	−0.0188 (7)	0.0078 (8)
C14	0.0228 (6)	0.0246 (6)	0.0205 (5)	−0.0097 (5)	0.0001 (4)	−0.0028 (4)
C15	0.0262 (6)	0.0240 (6)	0.0208 (5)	−0.0112 (5)	−0.0010 (4)	−0.0051 (4)
C16	0.0249 (6)	0.0226 (6)	0.0248 (5)	−0.0079 (5)	−0.0020 (4)	−0.0049 (4)
C17	0.0238 (6)	0.0244 (6)	0.0264 (5)	−0.0102 (5)	−0.0047 (4)	−0.0011 (4)
C18	0.0308 (6)	0.0284 (6)	0.0268 (5)	−0.0134 (5)	−0.0044 (5)	−0.0083 (5)
C19	0.0267 (6)	0.0263 (6)	0.0271 (6)	−0.0085 (5)	0.0007 (5)	−0.0096 (5)
C20	0.0231 (6)	0.0296 (6)	0.0234 (5)	−0.0094 (5)	0.0018 (4)	−0.0057 (5)
C21	0.0283 (6)	0.0324 (7)	0.0361 (6)	−0.0137 (6)	−0.0093 (5)	−0.0059 (5)
C22	0.0275 (6)	0.0302 (6)	0.0322 (6)	−0.0128 (5)	−0.0059 (5)	−0.0024 (5)
C23	0.0242 (6)	0.0305 (6)	0.0341 (6)	−0.0109 (5)	−0.0053 (5)	−0.0054 (5)
C24	0.0252 (6)	0.0260 (6)	0.0282 (6)	−0.0098 (5)	−0.0031 (5)	−0.0033 (5)
C25	0.0281 (6)	0.0322 (7)	0.0407 (7)	−0.0090 (5)	−0.0082 (5)	−0.0097 (5)
C26	0.0277 (7)	0.0405 (8)	0.0541 (8)	−0.0082 (6)	−0.0128 (6)	−0.0043 (6)
N1A	0.0143 (16)	0.0247 (19)	0.029 (2)	−0.0100 (10)	−0.0083 (6)	0.0066 (6)
C27A	0.0216 (16)	0.0215 (15)	0.042 (2)	−0.0101 (11)	−0.0085 (12)	0.0001 (10)
C28A	0.0240 (12)	0.0241 (11)	0.0294 (12)	−0.0065 (8)	−0.0043 (9)	−0.0056 (10)
C29A	0.0201 (10)	0.0213 (10)	0.0239 (11)	−0.0073 (8)	−0.0024 (9)	−0.0038 (9)
C30A	0.0278 (15)	0.0324 (13)	0.0263 (11)	−0.0109 (13)	−0.0036 (11)	−0.0048 (10)
C31A	0.0236 (17)	0.0367 (18)	0.0347 (15)	−0.0079 (16)	0.0031 (15)	0.0004 (11)
N2A	0.0147 (16)	0.0246 (19)	0.0227 (18)	−0.0086 (9)	−0.0066 (6)	−0.0006 (6)
C32A	0.0242 (17)	0.0328 (15)	0.0273 (15)	−0.0130 (13)	−0.0041 (11)	−0.0046 (9)
C33A	0.0233 (13)	0.0275 (12)	0.0253 (12)	−0.0085 (9)	−0.0014 (9)	−0.0070 (10)
C34A	0.0201 (11)	0.0227 (11)	0.0220 (11)	−0.0084 (9)	0.0014 (9)	−0.0036 (10)
C35A	0.0245 (16)	0.0275 (15)	0.0307 (13)	−0.0107 (14)	−0.0042 (11)	−0.0093 (11)
C36A	0.026 (2)	0.033 (2)	0.0403 (18)	−0.0095 (18)	0.0041 (15)	−0.0109 (14)
C37A	0.0157 (9)	0.0250 (9)	0.0315 (10)	−0.0057 (7)	−0.0020 (7)	−0.0027 (7)
C38A	0.0200 (10)	0.0263 (10)	0.0272 (9)	−0.0077 (7)	−0.0002 (7)	−0.0032 (7)

### (II) 4-(n-Hexyloxy)benzoic acid–(E)-1,2-bis(pyridin-4-yl)ethene Geometric parameters (Å, º)

**Table:**

O1—C7	1.3231 (16)	C24—C25	1.5152 (17)
O1—H1	0.99 (2)	C24—H24A	0.9900
O2—C7	1.2172 (15)	C24—H24B	0.9900
O3—C4	1.3660 (14)	C25—C26	1.5236 (18)
O3—C8	1.4336 (14)	C25—H25A	0.9900
O4—C20	1.3173 (15)	C25—H25B	0.9900
O4—H4	1.09 (2)	C26—H26A	0.9800
O5—C20	1.2155 (15)	C26—H26B	0.9800
O6—C17	1.3673 (14)	C26—H26C	0.9800
O6—C21	1.4342 (15)	N1A—C31A	1.319 (5)
C1—C2	1.3899 (17)	N1A—C27A	1.326 (5)
C1—C6	1.3929 (17)	C27A—C28A	1.367 (5)
C1—C7	1.4884 (16)	C27A—H27A	0.9500
C2—C3	1.3893 (16)	C28A—C29A	1.385 (3)
C2—H2	0.9500	C28A—H28A	0.9500
C3—C4	1.3893 (17)	C29A—C30A	1.395 (4)
C3—H3	0.9500	C29A—C37A	1.467 (3)
C4—C5	1.3939 (17)	C30A—C31A	1.391 (4)
C5—C6	1.3815 (17)	C30A—H30A	0.9500
C5—H5	0.9500	C31A—H31A	0.9500
C6—H6	0.9500	N2A—C32A	1.317 (5)
C8—C9	1.5079 (16)	N2A—C36A	1.324 (6)
C8—H8A	0.9900	C32A—C33A	1.381 (5)
C8—H8B	0.9900	C32A—H32A	0.9500
C9—C10	1.5198 (16)	C33A—C34A	1.392 (4)
C9—H9A	0.9900	C33A—H33A	0.9500
C9—H9B	0.9900	C34A—C35A	1.404 (4)
C10—C11	1.5250 (16)	C34A—C38A	1.468 (3)
C10—H10A	0.9900	C35A—C36A	1.381 (6)
C10—H10B	0.9900	C35A—H35A	0.9500
C11—C12	1.5161 (18)	C36A—H36A	0.9500
C11—H11A	0.9900	C37A—C38A	1.324 (3)
C11—H11B	0.9900	C37A—H37A	0.9500
C12—C13	1.517 (2)	C38A—H38A	0.9500
C12—H12A	0.9900	N1B—C31B	1.367 (11)
C12—H12B	0.9900	N1B—C27B	1.367 (12)
C13—H13A	0.9800	C27B—C28B	1.379 (10)
C13—H13B	0.9800	C27B—H27B	0.9500
C13—H13C	0.9800	C28B—C29B	1.373 (6)
C14—C19	1.3897 (16)	C28B—H28B	0.9500
C14—C15	1.3956 (17)	C29B—C30B	1.393 (6)
C14—C20	1.4857 (16)	C29B—C37B	1.458 (6)
C15—C16	1.3786 (16)	C30B—C31B	1.358 (8)
C15—H15	0.9500	C30B—H30B	0.9500
C16—C17	1.3918 (16)	C31B—H31B	0.9500
C16—H16	0.9500	N2B—C36B	1.355 (12)
C17—C18	1.3908 (18)	N2B—C32B	1.381 (12)
C18—C19	1.3920 (17)	C32B—C33B	1.374 (8)
C18—H18	0.9500	C32B—H32B	0.9500
C19—H19	0.9500	C33B—C34B	1.389 (6)
C21—C22	1.5119 (18)	C33B—H33B	0.9500
C21—H21A	0.9900	C34B—C35B	1.372 (6)
C21—H21B	0.9900	C34B—C38B	1.467 (7)
C22—C23	1.5181 (17)	C35B—C36B	1.342 (10)
C22—H22A	0.9900	C35B—H35B	0.9500
C22—H22B	0.9900	C36B—H36B	0.9500
C23—C24	1.5249 (17)	C37B—C38B	1.331 (6)
C23—H23A	0.9900	C37B—H37B	0.9500
C23—H23B	0.9900	C38B—H38B	0.9500
			
C7—O1—H1	112.0 (12)	C24—C23—H23B	109.0
C4—O3—C8	118.44 (9)	H23A—C23—H23B	107.8
C20—O4—H4	112.4 (12)	C25—C24—C23	113.04 (11)
C17—O6—C21	118.60 (10)	C25—C24—H24A	109.0
C2—C1—C6	118.87 (11)	C23—C24—H24A	109.0
C2—C1—C7	119.36 (11)	C25—C24—H24B	109.0
C6—C1—C7	121.77 (11)	C23—C24—H24B	109.0
C3—C2—C1	121.32 (11)	H24A—C24—H24B	107.8
C3—C2—H2	119.3	C24—C25—C26	113.35 (12)
C1—C2—H2	119.3	C24—C25—H25A	108.9
C4—C3—C2	118.90 (11)	C26—C25—H25A	108.9
C4—C3—H3	120.5	C24—C25—H25B	108.9
C2—C3—H3	120.5	C26—C25—H25B	108.9
O3—C4—C3	124.47 (11)	H25A—C25—H25B	107.7
O3—C4—C5	115.04 (11)	C25—C26—H26A	109.5
C3—C4—C5	120.49 (11)	C25—C26—H26B	109.5
C6—C5—C4	119.77 (11)	H26A—C26—H26B	109.5
C6—C5—H5	120.1	C25—C26—H26C	109.5
C4—C5—H5	120.1	H26A—C26—H26C	109.5
C5—C6—C1	120.64 (11)	H26B—C26—H26C	109.5
C5—C6—H6	119.7	C31A—N1A—C27A	115.7 (4)
C1—C6—H6	119.7	N1A—C27A—C28A	125.5 (5)
O2—C7—O1	123.31 (11)	N1A—C27A—H27A	117.2
O2—C7—C1	123.23 (12)	C28A—C27A—H27A	117.2
O1—C7—C1	113.45 (11)	C27A—C28A—C29A	118.6 (3)
O3—C8—C9	106.79 (10)	C27A—C28A—H28A	120.7
O3—C8—H8A	110.4	C29A—C28A—H28A	120.7
C9—C8—H8A	110.4	C28A—C29A—C30A	117.20 (19)
O3—C8—H8B	110.4	C28A—C29A—C37A	118.8 (2)
C9—C8—H8B	110.4	C30A—C29A—C37A	124.0 (2)
H8A—C8—H8B	108.6	C31A—C30A—C29A	118.6 (3)
C8—C9—C10	113.37 (10)	C31A—C30A—H30A	120.7
C8—C9—H9A	108.9	C29A—C30A—H30A	120.7
C10—C9—H9A	108.9	N1A—C31A—C30A	124.2 (4)
C8—C9—H9B	108.9	N1A—C31A—H31A	117.9
C10—C9—H9B	108.9	C30A—C31A—H31A	117.9
H9A—C9—H9B	107.7	C32A—N2A—C36A	117.6 (5)
C9—C10—C11	112.37 (10)	C32A—N2A—H4	120.6 (9)
C9—C10—H10A	109.1	C36A—N2A—H4	121.7 (9)
C11—C10—H10A	109.1	N2A—C32A—C33A	123.5 (4)
C9—C10—H10B	109.1	N2A—C32A—H32A	118.2
C11—C10—H10B	109.1	C33A—C32A—H32A	118.2
H10A—C10—H10B	107.9	C32A—C33A—C34A	119.4 (3)
C12—C11—C10	113.19 (11)	C32A—C33A—H33A	120.3
C12—C11—H11A	108.9	C34A—C33A—H33A	120.3
C10—C11—H11A	108.9	C33A—C34A—C35A	116.8 (2)
C12—C11—H11B	108.9	C33A—C34A—C38A	120.3 (2)
C10—C11—H11B	108.9	C35A—C34A—C38A	122.9 (3)
H11A—C11—H11B	107.8	C36A—C35A—C34A	118.6 (3)
C11—C12—C13	113.36 (13)	C36A—C35A—H35A	120.7
C11—C12—H12A	108.9	C34A—C35A—H35A	120.7
C13—C12—H12A	108.9	N2A—C36A—C35A	123.9 (5)
C11—C12—H12B	108.9	N2A—C36A—H36A	118.0
C13—C12—H12B	108.9	C35A—C36A—H36A	118.0
H12A—C12—H12B	107.7	C38A—C37A—C29A	127.8 (2)
C12—C13—H13A	109.5	C38A—C37A—H37A	116.1
C12—C13—H13B	109.5	C29A—C37A—H37A	116.1
H13A—C13—H13B	109.5	C37A—C38A—C34A	125.7 (2)
C12—C13—H13C	109.5	C37A—C38A—H38A	117.1
H13A—C13—H13C	109.5	C34A—C38A—H38A	117.1
H13B—C13—H13C	109.5	C31B—N1B—C27B	117.7 (10)
C19—C14—C15	118.54 (11)	N1B—C27B—C28B	119.9 (10)
C19—C14—C20	120.46 (11)	N1B—C27B—H27B	120.0
C15—C14—C20	120.99 (11)	C28B—C27B—H27B	120.0
C16—C15—C14	121.05 (11)	C29B—C28B—C27B	122.4 (6)
C16—C15—H15	119.5	C29B—C28B—H28B	118.8
C14—C15—H15	119.5	C27B—C28B—H28B	118.8
C15—C16—C17	119.71 (11)	C28B—C29B—C30B	117.0 (4)
C15—C16—H16	120.1	C28B—C29B—C37B	123.8 (5)
C17—C16—H16	120.1	C30B—C29B—C37B	119.2 (5)
O6—C17—C18	124.65 (11)	C31B—C30B—C29B	119.9 (5)
O6—C17—C16	114.96 (11)	C31B—C30B—H30B	120.1
C18—C17—C16	120.38 (11)	C29B—C30B—H30B	120.1
C17—C18—C19	119.06 (11)	C30B—C31B—N1B	123.1 (8)
C17—C18—H18	120.5	C30B—C31B—H31B	118.5
C19—C18—H18	120.5	N1B—C31B—H31B	118.5
C14—C19—C18	121.24 (11)	C36B—N2B—C32B	116.8 (10)
C14—C19—H19	119.4	C33B—C32B—N2B	121.6 (8)
C18—C19—H19	119.4	C33B—C32B—H32B	119.2
O5—C20—O4	123.15 (11)	N2B—C32B—H32B	119.2
O5—C20—C14	123.36 (11)	C32B—C33B—C34B	119.8 (6)
O4—C20—C14	113.49 (10)	C32B—C33B—H33B	120.1
O6—C21—C22	106.50 (11)	C34B—C33B—H33B	120.1
O6—C21—H21A	110.4	C35B—C34B—C33B	117.5 (5)
C22—C21—H21A	110.4	C35B—C34B—C38B	118.5 (5)
O6—C21—H21B	110.4	C33B—C34B—C38B	123.9 (5)
C22—C21—H21B	110.4	C36B—C35B—C34B	121.4 (7)
H21A—C21—H21B	108.6	C36B—C35B—H35B	119.3
C21—C22—C23	113.34 (11)	C34B—C35B—H35B	119.3
C21—C22—H22A	108.9	C35B—C36B—N2B	122.6 (10)
C23—C22—H22A	108.9	C35B—C36B—H36B	118.7
C21—C22—H22B	108.9	N2B—C36B—H36B	118.7
C23—C22—H22B	108.9	C38B—C37B—C29B	125.2 (4)
H22A—C22—H22B	107.7	C38B—C37B—H37B	117.4
C22—C23—C24	112.77 (11)	C29B—C37B—H37B	117.4
C22—C23—H23A	109.0	C37B—C38B—C34B	128.2 (4)
C24—C23—H23A	109.0	C37B—C38B—H38B	115.9
C22—C23—H23B	109.0	C34B—C38B—H38B	115.9
			
C6—C1—C2—C3	0.13 (16)	C31A—N1A—C27A—C28A	0.6 (9)
C7—C1—C2—C3	−179.81 (9)	N1A—C27A—C28A—C29A	−0.5 (7)
C1—C2—C3—C4	0.25 (16)	C27A—C28A—C29A—C30A	0.0 (4)
C8—O3—C4—C3	−3.85 (15)	C27A—C28A—C29A—C37A	179.2 (3)
C8—O3—C4—C5	176.62 (10)	C28A—C29A—C30A—C31A	0.3 (4)
C2—C3—C4—O3	−179.65 (9)	C37A—C29A—C30A—C31A	−178.9 (3)
C2—C3—C4—C5	−0.14 (16)	C27A—N1A—C31A—C30A	−0.2 (9)
O3—C4—C5—C6	179.21 (10)	C29A—C30A—C31A—N1A	−0.2 (7)
C3—C4—C5—C6	−0.35 (17)	C36A—N2A—C32A—C33A	−3.3 (10)
C4—C5—C6—C1	0.74 (17)	N2A—C32A—C33A—C34A	2.1 (7)
C2—C1—C6—C5	−0.63 (16)	C32A—C33A—C34A—C35A	0.4 (4)
C7—C1—C6—C5	179.31 (10)	C32A—C33A—C34A—C38A	−178.8 (3)
C2—C1—C7—O2	8.78 (16)	C33A—C34A—C35A—C36A	−1.5 (5)
C6—C1—C7—O2	−171.16 (10)	C38A—C34A—C35A—C36A	177.7 (4)
C2—C1—C7—O1	−171.90 (9)	C32A—N2A—C36A—C35A	2.1 (11)
C6—C1—C7—O1	8.17 (15)	C34A—C35A—C36A—N2A	0.3 (10)
C4—O3—C8—C9	−175.16 (9)	C28A—C29A—C37A—C38A	−175.75 (18)
O3—C8—C9—C10	178.95 (9)	C30A—C29A—C37A—C38A	3.4 (3)
C8—C9—C10—C11	179.73 (9)	C29A—C37A—C38A—C34A	−179.43 (16)
C9—C10—C11—C12	−177.26 (10)	C33A—C34A—C38A—C37A	−176.93 (18)
C10—C11—C12—C13	176.62 (10)	C35A—C34A—C38A—C37A	4.0 (3)
C19—C14—C15—C16	0.84 (15)	C31B—N1B—C27B—C28B	−3 (2)
C20—C14—C15—C16	−177.99 (9)	N1B—C27B—C28B—C29B	3.9 (17)
C14—C15—C16—C17	−1.61 (15)	C27B—C28B—C29B—C30B	−2.1 (10)
C21—O6—C17—C18	−3.71 (15)	C27B—C28B—C29B—C37B	177.1 (8)
C21—O6—C17—C16	176.94 (9)	C28B—C29B—C30B—C31B	0.1 (9)
C15—C16—C17—O6	−179.76 (9)	C37B—C29B—C30B—C31B	−179.2 (6)
C15—C16—C17—C18	0.86 (15)	C29B—C30B—C31B—N1B	0.3 (14)
O6—C17—C18—C19	−178.68 (10)	C27B—N1B—C31B—C30B	1 (2)
C16—C17—C18—C19	0.64 (16)	C36B—N2B—C32B—C33B	5 (2)
C15—C14—C19—C18	0.70 (15)	N2B—C32B—C33B—C34B	−3.4 (14)
C20—C14—C19—C18	179.53 (10)	C32B—C33B—C34B—C35B	0.5 (8)
C17—C18—C19—C14	−1.43 (16)	C32B—C33B—C34B—C38B	−178.1 (5)
C19—C14—C20—O5	−2.65 (16)	C33B—C34B—C35B—C36B	0.4 (10)
C15—C14—C20—O5	176.15 (11)	C38B—C34B—C35B—C36B	179.0 (7)
C19—C14—C20—O4	177.81 (9)	C34B—C35B—C36B—N2B	1.7 (17)
C15—C14—C20—O4	−3.39 (14)	C32B—N2B—C36B—C35B	−5 (2)
C17—O6—C21—C22	−179.01 (9)	C28B—C29B—C37B—C38B	−3.2 (6)
O6—C21—C22—C23	175.75 (9)	C30B—C29B—C37B—C38B	175.9 (4)
C21—C22—C23—C24	178.72 (9)	C29B—C37B—C38B—C34B	179.3 (3)
C22—C23—C24—C25	176.43 (9)	C35B—C34B—C38B—C37B	−168.6 (4)
C23—C24—C25—C26	175.91 (10)	C33B—C34B—C38B—C37B	10.0 (7)

### (II) 4-(n-Hexyloxy)benzoic acid–(E)-1,2-bis(pyridin-4-yl)ethene Hydrogen-bond geometry (Å, º)

*Cg*1 and *Cg*2 are the centroids of the benzene C1–C6 and C14–C19 rings, respectively.

**Table:**

*D*—H···*A*	*D*—H	H···*A*	*D*···*A*	*D*—H···*A*
O1—H1···N1*A*	0.99 (2)	1.65 (2)	2.635 (5)	176.5 (15)
O1—H1···N1*B*	0.99 (2)	1.63 (2)	2.616 (14)	176.3 (19)
O4—H4···N2*A*	1.08 (3)	1.51 (3)	2.584 (6)	172.8 (18)
O4—H4···N2*B*	1.08 (3)	1.54 (3)	2.618 (15)	172.5 (18)
C12—H12*A*···*Cg*1^i^	0.99	2.99	3.720 (2)	132
C24—H24*A*···*Cg*2^ii^	0.99	2.93	3.838 (2)	154

Symmetry codes: (i) *x*+1, *y*, *z*; (ii) *x*−1, *y*, *z*.
